# Endothelial keratoplasty: The relationship between recipient anterior chamber depth and endothelial cell loss

**DOI:** 10.1097/MD.0000000000016171

**Published:** 2019-06-21

**Authors:** Asem A. Alqudah, Alex J. Bauer, Michael Straiko, Mark A. Terry

**Affiliations:** a Devers Eye Institute; b Lions VisionGift, Portland, Oregon; c Jordan University of Science and Technology (JUST), Irbid, Jordan.

**Keywords:** anterior chamber depth, DSAEK, endothelial cell loss

## Abstract

**Purpose:**

To determine the relationship between anterior chamber depth (ACD) and percent endothelial cell loss (ECL) after Descemet Stripping Automated Endothelial Keratoplasty (DSAEK).

**Method:**

In 78 eyes receiving triple procedure (DSAEK combined with cataract extraction and posterior chamber intraocular lens (PCIOL) implantation), ACD was measured preoperatively with an intraocular lens (IOL) Master and ECL was calculated with specular microscopy at 6 months, 1, 2, 3, and 4 years postoperatively. ACD and ECL from all 78 eyes were compared using correlation analysis and students *t* test. Eyes were then separated into 2 groups based on ACD, group 1 with ACD < 3mm and group 2 with ACD ≥ 3mm. Students *t* test was then performed to compare group 1 and group 2 ECL at 6 months, 1, 2, 3, and 4 years postoperative.

**Results:**

Mean ACD for all 78 eyes was 2.93 ± 0.43 mm. Mean ECL was 32.7%, 27.6%, 29.6%, 32.5%, and 37.2% at 6 months, 1, 2, 3, and 4 years. No significant correlation between ACD and ECL was observed at any time point for the combined analysis of 78 eyes (*P* > .05). At 2 and 4 years postoperative, mean ECL was 32.6% ± 16.1% and 43.0% ± 23.2% in eyes with ACD < 3mm and 25.3% ± 13.0% and 29.6% ± 18.2% in eyes with ACD ≥ 3 mm (*P* = .041 at 2 years and .008 at 4 years).

**Conclusion:**

ACD and ECL were not directly correlated; however, there may be a threshold ACD in which shallower anterior chambers preoperatively result in greater donor ECL over time.

## 1 Introduction

Endothelial Keratoplasty (EK) is the most widely performed procedure in the United States for patients with corneal endothelial dysfunction such as Fuchs’ dystrophy and pseudophakic bullous keratopathy. Although Descemet Membrane Endothelial Keratoplasty (DMEK) has been rapidly evolving, Descemet Stripping Automated Endothelial Keratoplasty (DSAEK) is still the most commonly performed technique for EK.^[1]^

Many previous articles have studied the factors that may affect the graft survival in DSAEK, including donor tissue size,^[2]^ donor age,^[3]^ donor tissue storage time,^[4]^ and donor tissue preoperative endothelial cell count.^[5]^ No significant correlations were found between overall graft survival and any of these factors.

There are currently many DSAEK techniques for donor tissue insertion and manipulation. Insertion of the graft using various forceps remains a common method of tissue insertion, and a shallow anterior chamber can make this maneuver difficult. In addition, once the DSAEK graft is delivered to the anterior chamber, a shallow chamber can make complete unfolding and positioning more difficult. The greater the manipulation of donor tissue, the greater the risk of endothelial damage.^[6,7]^

In this report, we studied the relationship between anterior chamber depth (ACD) and donor Endothelial Cell Loss (ECL) for the short and long-term postoperative periods in a group of DSAEK patients who underwent triple procedures. To our knowledge, this would be the first article that studies this relationship.

## 2 Methods

### 2.1 Protocol

This is part of a retrospective series of the EK patient registry at Devers Eye Institute, and a continuation of our prospective study of EK. A Legacy Health Institutional Review Board approved and Health Insurance Portability and Accountability Act compliant clinical protocol and surgical consent form was developed and enrollment was initiated for patients with endothelial dysfunction.

No specific requests were made of the eye bank to provide tissue for EK with any different characteristics than what is normally requested for full thickness PK tissues. Donor tissues with an endothelial cell density (ECD) more than 2000 cells/mm^2^, any age between 4 and 75 years old, and death to transplantation time of up to 12 days were accepted.

Patients receiving a triple procedure for Fuchs’ endothelial dystrophy and cataract were considered eligible for the study.

Among the 78 triple procedure cases performed between October 20, 2005 and February 27, 2008, 65 cases in 45 patients had complete datasets available for analysis.

Eyes with a history of age-related macular degeneration, cystoid macular edema, controlled glaucoma, and other comorbidities were not excluded as visual acuity was not an outcome of the study.

Cases with glaucoma drainage devices, retained anterior chamber intraocular lens (IOLs), graft dislocation or other possible causes of endothelial damage were identified and excluded from analysis.

The ACD for all patients were measured preoperatively with the IOL Master instrument.

### 2.2 Specular microscopy data

The vast majority of donor tissue came from Lions VisionGift in Portland, Oregon. Specular images were obtained with an EB-3000 XYZ Eyebank specular microscope (HAI Laboratories, Inc., Lexington, MA). The preoperative cell counts were obtained using an apices digitized method and the manufacturer's calibrations for magnification. The apices of at least 100 cells from the endothelial images of each cornea were counted. Preoperative donor tissue specular microscopy was performed by 3 trained eye bank technicians, all of whom had at least 1 year of experience with this technology.

Postoperative specular microscopy measurements of ECD were acquired at Devers Eye Institute using the Konan SP4000 noncontact specular microscope (Konan Medical Corp., Fairlawn, NJ) at 6 months, 1 year, then annually after surgery. A certified ophthalmic technician (COT) performed all postoperative testing of patients using the same specular microscope each time.

These postoperative cell counts were obtained using the manufacturer's calibrations for magnification and were counted with a fixed-frame method with the protocol requiring the marking of at least 50 to 100 cells for each image. Specular microscopy measurements with insufficient quality of the image were not included in the analysis. Insufficient quality of the image was determined subjectively by the examining physician based on the edge clarity of the individual cells. Approximately 8% of specular images are rejected in the authors’ clinical program, and approximately 10% of the time central specular images cannot be obtained despite a crystal-clear graft. Clarity of the graft was not an issue with any rejected specular image. Analysis of endothelial pleomorphism and polymegathism was not performed in this study.

### 2.3 Surgical procedure

In all cases of our triple procedures, the phacoemulsification and intraocular lens implantation were done before DSAEK through the same 5 mm temporal scleral tunnel, but with only a 2.8 mm keratome opening into the AC. This opening was subsequently enlarged to the full 5 mm for the insertion of the donor tissue.

DSAEK was performed with a standard technique as previously published.^[8,9]^ In all cases, the tissue was precut by an eye bank technician and then provided to the surgeon, usually within 26 hours of precutting.^[3,8,10]^ A video of the DSAEK technique using precut donor tissue is available online (https://www.youtube.com/watch?v=mtu8dxZUCx0) from a previous report on the use of precut tissue in DSAEK.

In brief, the standard DSAEK procedure involves placement of 2 limbal 1-mm wide paracentesis incisions on either side of a 5-mm temporal scleral access wound. This wound is placed approximately 0.5 mm peripheral to the temporal limbus with a scleral– corneal tunnel of approximately 2 mm until entry into the anterior chamber. After filling the anterior chamber with cohesive viscoelastic, a circular template mark is placed on the surface of the cornea to delineate the area of stripping, and this determines the same size for the donor tissue. The donor diameter chosen is individualized for each patient and is based on the largest diameter circle that can be fit on the individual cornea without overlap of the edge of the tissue over the 2 paracentesis sites or the corneal portion of the main insertion wound. After stripping of the recipient Descemet's membrane and scraping of the recipient bed with a Terry Scraper (Bausch & Lomb Surgical, St. Louis, MO), the Healon (Abbott Medical Optics, Santa Ana, CA) viscoelastic is removed from the eye with standard automated irrigation and aspiration technique. The pupil is constricted with Miochol-E (Novartis Pharmaceuticals Corporation, Basel, Switzerland), the microscope is moved to the donor table, and the donor tissue is trephinated with the same size diameter as the recipient template mark. After preparation, the donor tissue is grasped and then inserted with non-coapting Charlie II insertion forceps (Bausch & Lomb Surgical) in a taco configuration with the 60% edge placed anteriorly in the chamber. The tissue is unfolded with deepening of the anterior chamber with balanced salt solution and injection of air to complete unfolding of the tissue into position. After surface sweeping with the Cindy Sweeper (Bausch & Lomb Surgical) to remove interface fluid while the anterior chamber is filled with air, the procedure is completed with complete removal of the air from the chamber and then reinjection of a specified amount of air to yield only a 5 or 6 mm residual bubble for minimal graft support. This small air bubble is preferred to avoid the event of pupillary block that can occur with larger air-bubble retention at the conclusion of surgery.

Surgery was performed by 5 corneal surgeons at Devers Eye Institute, with the senior surgeon (MAT) performing 63% of the cases. All surgeons used the exact same surgical technique for every case.

### 2.4 Statistical analysis

Postoperative ECD and percentage ECL was recorded at 6 months, 1 year, 2 years, 3 years and 4 years. The preoperative ACD and the postoperative ECD at each postoperative time point were first compared using Pearson correlation analysis to see if there was any direct relationship between the preoperative ACD and ECL at different time intervals. Then the cases were divided into 2 groups based upon the ACD. Group 1 had a preoperative ACD < 3mm (45 eyes) and group 2 had a preoperative ACD ≥ 3 mm (33 eyes). Baseline and intraoperative characteristics were compared between groups using independent samples *t* test and Chi Square test as appropriate. All statistics were performed using SPSS v. 23 (IBM, Chicago, IL).

## 3 Results

The 78 eyes in this retrospective study were from 49 patients with a mean age at surgery of 63.5 ± 8.7 years (range 42–83 years). 54 eyes (69.2%) were from females. All required DSAEK surgery due to Fuchs’ endothelial dystrophy.

Baseline characteristics that were evaluated and compared include mean donor age, tissue preservation time, preoperative ECD, and number of tissues that came from donors with a history of diabetes. Group 1 and group 2 were not statistically different for mean donor age (54.9 ± 15.0 years vs 55.5 ± 13.0 years, *P* = .848), tissue preservation time (3.5 ± 1.3 days vs 3.5 ± 1.3 days, *P* = .952), preoperative ECD (2939.2 ± 396.5 cells/mm^2^ vs 2879.3 ± 291.0 cells/mm^2^, *P* = .465), or donor diabetes (8/45 eyes vs 11/33 eyes, *P* = .114).

Patient demographics did not differ between groups with respect to gender or surgeon experience.

**Figure 1:**
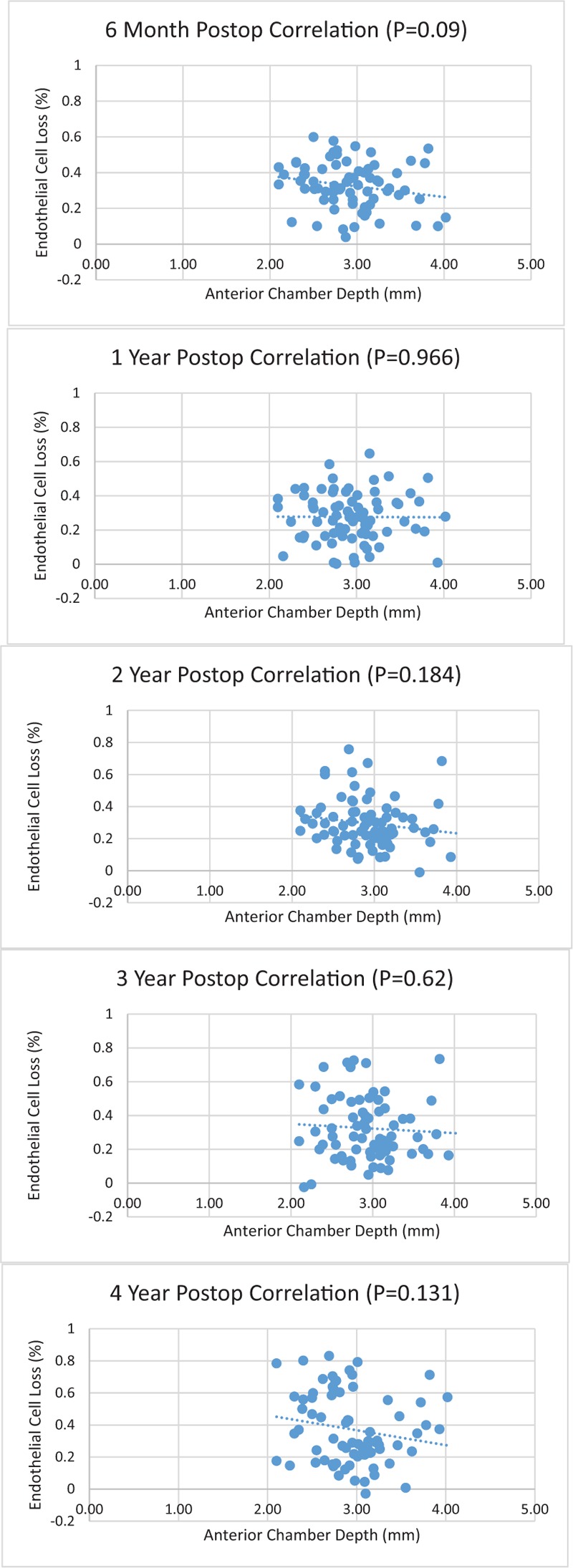
Pearson correlation analysis revealed no statistical significance comparing anterior chamber depth and endothelial cell loss at different post-operative time points.

The mean ACD for the combined groups (78 eyes) was 2.93 ± 0.43 mm (range 2.10–4.02 mm). The mean percent ECL from baseline for the combined groups was 32.7% ± 13.0% at 6 months, 27.6% ± 14.3% at 1 year, 29.6% ± 15.2% at 2 years, 32.5% ± 18.8% at 3 years, and 37.2% ± 22.1% at 4 years. The ACD for all eyes did not correlate significantly with ECL at any postoperative time point (6 months: *r* = −0.197, *P* = .09, 1 year: *r* = −0.006, *P* = .966, 2 years: *r* = −0.155, *P* = .184, 3 years: *r* = −0.06, *P* = .62, 4 years: *r* = −0.176, *P* = .131) (Fig. 1).

**Figure 2:**
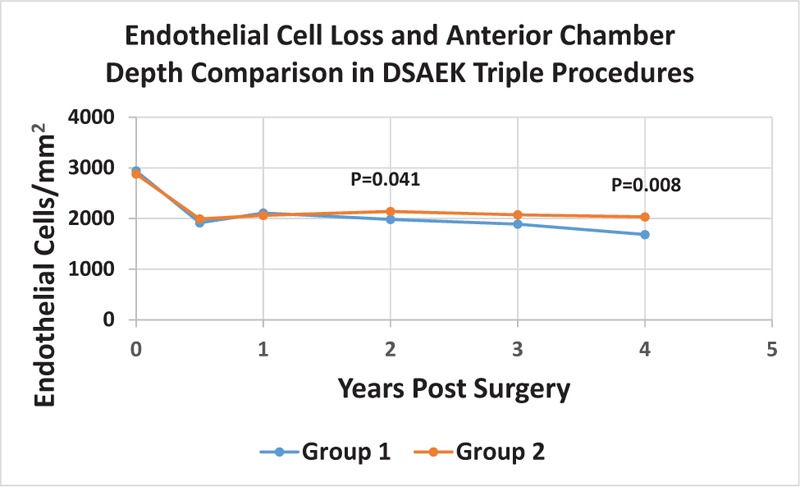
compares ECL for Group 1 (ACD < 3mm) and Group 2 (ACD ≥ 3mm) at each time point. Statistical significance was reached at year 2 (*P* = .041) and 4 (*P* = .008).

Mean ACD was 2.65 ± 0.25 mm for Group 1 and 3.32 ± 0.29 mm for group 2. Mean percentages of ECL for group 1 were 34.5% ± 13.1% at 6 months, 27.4% ± 14.4% at 1 year, 32.6% ± 16.1% at 2 years, 35.4% ± 20.6% at 3 years and 43.0% ± 23.2% at 4 years. For group 2, the percentages were 30.0% ± 12.5%, 27.9% ± 14.4%, 25.3% ± 13.0%, 28.5% ± 15.6%, 29.6% ± 18.2% at 6 months, 1 year, 2 years, 3 years and 4 years, respectively. Table 1 The *P* values for the 2-group comparison were (6 months: *P* = .146, 1 year: *P* = .891, 2 years: *P* = .041, 3 years: *P* = .124 and 4 years: *P* = .008). The difference between the 2 groups at 2 and 4 years was statistically significant (*P* = .041 and .008). Figure 2

**Table 1:**
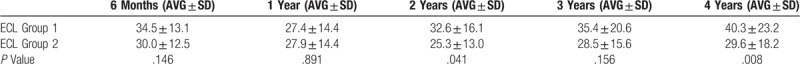
Average endothelial cell loss at each postoperative visit and corresponding *t* test comparison.

## 4 Discussion

One of the aims of corneal grafts, in general, is to provide the patient with the best possible visual acuity for the longest period of time. Early and late graft failures are reported complications of EK that usually require repeat surgery.

Delivering the largest number of healthy donor endothelial cells into the recipient's eye is the main goal during the DSAEK surgery. This is usually accomplished by choosing donor tissues with an excellent endothelial cell count and by the meticulous handling of the donor tissue during the surgery.

The relationship between preoperative donor endothelial cell count and the overall graft survival has been previously studied.^[5]^ No significant correlation has been found between the preoperative endothelial cell count and the graft survival, given that all donor tissues had ECD higher than 2000 cell/mm^2^.

The immediate ECL after DSAEK has also been studied.^[11,12]^ The immediate endothelial loss in a wet lab experiment performed at the eye bank was found to be about 18% with a significant central cell loss related to tissue grasping and folding.^[11]^ The earliest postoperative specular microscopy done in our center is at the 6 months postoperative time point. This count represents a combination of both the intraoperative ECL as well as the cell loss in the first 6 months postoperatively.

In this study, the ECL was similar between the 2 ACD comparison groups at the 6 month and 1 year postoperative visits. However, by 2 years postoperatively, the difference in ECL was statistically significant and this difference increased even more by year 4. It may be that the surgical trauma due to dealing with a shallow ACD at the time of surgery has a delayed and deleterious effect on the long-term survival of the donor endothelium.

One of the limitations of this study is that in combined phacoemulsification, IOL placement, and DSAEK surgery, the measured preoperative ACD may differ from the postoperative ACD. However, with the exception of severe nuclear sclerosis eyes (which none of this series of cases contained), it was found that deeper ACD before cataract surgery is associated with deeper ACD after surgery, which keeps the relative difference in ACD between the 2 groups.^[13]^

As a part of the management in patients that will undergo triple DSAEK procedures, taking note of the preop ACD may be an important factor in predicting the long term ECL of the DSAEK graft.

## Author contributions

**Conceptualization:** Asem A. Alqudah.

**Data curation:** Alex J. Bauer.

**Formal analysis:** Alex J. Bauer.

**Investigation:** Asem A. Alqudah, Alex J. Bauer.

**Methodology:** Alex J. Bauer.

**Software:** Asem A. Alqudah, Alex J. Bauer.

**Supervision:** Michael Straiko, Mark A. Terry.

**Writing – original draft:** Asem A. Alqudah.

**Writing – review & editing:** Asem A. Alqudah, Alex J. Bauer, Mark A. Terry.
